# The Aged Striatum: Evidence of Molecular and Structural Changes Using a Longitudinal Multimodal Approach in Mice

**DOI:** 10.3389/fnagi.2022.795132

**Published:** 2022-01-24

**Authors:** Bruno Lima Giacobbo, Özgün Özalay, Tomas Mediavilla, Madelene Ericsson, Jan Axelsson, Anna Rieckmann, Fahad Sultan, Daniel Marcellino

**Affiliations:** ^1^Department of Integrative Medical Biology, Umeå University, Umeå, Sweden; ^2^Umeå Centre for Molecular Medicine, Umeå University, Umeå, Sweden; ^3^Department of Radiation Sciences, Umeå University, Umeå, Sweden

**Keywords:** PET, dopamine, structural MRI, aging, senescence, VBM

## Abstract

To study the aging human brain requires significant resources and time. Thus, mice models of aging can provide insight into changes in brain biological functions at a fraction of the time when compared to humans. This study aims to explore changes in dopamine D_1_ and D_2_ receptor availability and of gray matter density in striatum during aging in mice and to evaluate whether longitudinal imaging in mice may serve as a model for normal brain aging to complement cross-sectional research in humans. Mice underwent repeated structural magnetic resonance imaging (sMRI), and [^11^C]Raclopride and [^11^C]SCH23390 positron emission tomography (PET) was performed on a subset of aging mice. PET and sMRI data were analyzed by binding potential (BP_*ND*_), voxel- and tensor-based morphometry (VBM and TBM, respectively). Longitudinal PET revealed a significant reduction in striatal BP_*ND*_ for D_2_ receptors over time, whereas no significant change was found for D_1_ receptors. sMRI indicated a significant increase in modulated gray matter density (mGMD) over time in striatum, with limited clusters showing decreased mGMD. Mouse [^11^C]Raclopride data is compatible with previous reports in human cross-sectional studies, suggesting that a natural loss of dopaminergic D_2_ receptors in striatum can be assessed in mice, reflecting estimates from humans. No changes in D_1_ were found, which may be attributed to altered [^11^C]SCH23390 kinetics in anesthetized mice, suggesting that this tracer is not yet able to replicate human findings. sMRI revealed a significant increase in mGMD. Although contrary to expectations, this increase in modulated GM density may be attributed to an age-related increase in non-neuronal cells.

## Introduction

Aging affects virtually every physiological process within all organs of the human body (Lopez-Otin et al., 2013). In the brain, time affects the development of neuronal circuitry as organisms mature (Gilmore et al., 2018). After reaching its peak, however, the brain reaches a period of senescence, when a natural deterioration of biological function occurs. Such deterioration is reflected by a general reduction in cognitive performance at older ages when compared with younger adults, as well as an increased risk factor for many brain-related disorders (Aunan et al., 2016; Siman-Tov et al., 2016). In that regard, non-invasive imaging techniques such as Positron Emission Tomography (PET) and Magnetic Resonance Imaging (MRI) allow researchers to explore structural, functional, and molecular integrities of the brain to further understand age-related changes using a longitudinal approach (Papenberg et al., 2019), and to determine how such changes relate to altered human behavior and cognitive performance (Nyberg, 2017).

Cross-sectional PET studies in humans have demonstrated a robust impact of age on striatal dopamine system activity through D_1_ and D_2_ dopamine receptor binding potential (BP_*ND*_), where both receptor subtypes show reduced BP_*ND*_ with age, while dopaminergic synthesis was found to remain relatively stable (Karrer et al., 2017). In addition, morphological and functional changes are also observed in the human brain over time (Nyberg et al., 2010). These results highlight the importance of dopaminergic signaling for maintenance of optimal cognitive function and that reductions in dopamine receptor availability might be responsible for age-related cognitive decline (Backman et al., 2006; Lovden et al., 2018; Juarez et al., 2019; Karalija et al., 2021). However, most of the previous studies of aging on dopaminergic systems are largely based on cross-sectional data (Nordin et al., 2021). This is not optimal since studies of functional and structural imaging markers have shown that cross-sectional data may be affected by selection and cohort effects and deviate from estimates derived from longitudinal data (Nyberg et al., 2010). For the study of molecular targets, longitudinal comparisons are still rare because repeated PET studies require repeated exposure to radiation, in addition to limitations due to high cost and the long human lifespan (Nevalainen et al., 2015). To overcome the difficulties associated with longitudinal imaging in humans, recent studies have proposed complementing human research with longitudinal studies of aging in animal models (Mitchell et al., 2015; McQuail et al., 2020). Rodents, such as mice and rats, present similar molecular and structural patterns in the brain as humans, and their shorter lifespan (e.g., mice live between 2.5 and 3 years) make such animals an appropriate model for understanding the basic molecular processes related to aging in a longitudinal fashion.

With this overall goal in mind, the aim of this study was to estimate age-related changes in both molecular (D_1_ and D_2_ receptor availability) and structural (gray matter density) components in mice using repeated non-invasive imaging across the lifespan. Dynamic PET scans for dopamine receptor binding and structural MRI were performed over the course of 12 months, encompassing an extended period of senescence in aged mice. Imaging methods and analyses were aligned with those in human research. The working hypothesis of this study, based on reported human observations, is that both gray matter density—evaluated by sMRI—and dopamine D_1_ and D_2_ receptor availability—measured by PET using both [^11^C]SCH23390 and [^11^C]Raclopride—would decrease with age in mouse striatum [comprised of caudate/putamen (CPu) and nucleus accumbens (NAc)], consistent with the findings reported in human studies (Karrer et al., 2017). Such results would confirm that longitudinal imaging research of the dopamine system and gray matter in mice would be a useful translational approach to understand the aging brain.

## Materials and Methods

### Animals and Study Design

Six-month-old male C57Bl/6J mice (*n* = 22; Aged Black 6 stock no: 000664; Jackson Laboratory, California, United States) were purchased and housed at Umeå Centre for Comparative Biology (UCCB) using standard conditions (temperature: 21°C; 12 h/12 h dark/light cycle; water and food *ad libitum*). Mice were monitored each week from 11 months of age for the entire experiment to repeatedly handle and weigh each individual (Supplementary Figure 1). All procedures were approved by the regional Animal Research Ethics Committee of Northern Norrland and by the Swedish Board of Agriculture (permit: A17-2019). All animals underwent structural MR examination at 12, 18, and 24 months of age and a subset of these individuals (*n* = 9) also underwent longitudinal PET scans as follows: [^11^C]Raclopride at 12, 18, and 24 months of age; and [^11^C]SCH23390 at 15, 18, and 21 months of age (*n* = 8). Animals with only structural MRI had a total of three imaging sessions over the 12-month period, while animals with both MRI and PET had a total of nine sessions (Supplementary Figure 2). Due to a failed scan at month 18 for [^11^C]Raclopride, three additional animals were included for D_2_ PET at that timepoint. In addition, two additional animals were scanned for [^11^C]Raclopride at 24 months from the MRI only group. Due to the observational characteristic of this study, and similar genetic background for all additional animals, any inter-individual variability was relatively low, and accounted for in the statistical procedures. A table with the sample size at all timepoints can be found in Supplementary Table 1.

### Positron Emission Tomography Data Acquisition

PET data was acquired equally for both [^11^C]SCH23390 and [^11^C]Raclopride. Animals were anesthetized using 4% isoflurane/O_2_ and maintained at 1.5–2% isoflurane/O_2_ throughout the remainder of the procedure. A cannula was placed into the tail vein of the animal and flushed with heparinized saline. Animals were placed into the PET/CT scanner (nanoScan^®^PET/CT system, Mediso, Hungary) and a 5-min 3D-computerized tomography (CT) was acquired to generate a material map for attenuation correction, followed by a 60-min acquisition with a 1-min injection of either [^11^C]Raclopride or [^11^C]SCH23390, and simultaneous data acquisition from the start of injection (Mean injected dose: 18.11 ± 4.17 MBq and 16.27 ± 4.20 MBq for [^11^C]Raclopride and [^11^C]SCH23390, respectively). Prior to the injection of radiotracer, the vial containing the total amount of radiotracer was heated at 80°C for 5 min under a constant airflow to evaporate ethanol content derived from radiotracer production. At the end of the scan the mouse was placed into a cage to recover from anesthesia before returned to its home cage. Throughout all PET experiments, respiration and temperature were constantly monitored.

### Positron Emission Tomography Data Reconstruction and Analysis

PET reconstruction of images for all radiotracers was performed using a Maximum Likelihood Expectation-Maximization (MLEM) with 4 subsets and 4 iterations. The resulting reconstructed data was binned into 43 frames (12 × 10 s; 6 × 20 s; 6 × 40 s; 6 × 80 s; 6 × 160 s; 7 × 240 s). The data was normalized for attenuation, scatter and radioactive decay and the resulting image was 200 × 200 × 250 voxels with a voxel size of 0.2 mm, with brain centered in the FOV. Reconstructed images were aligned and averaged to the first 15 min of the scan and averaged images were coregistered to create study-specific templates for both [^11^C]Raclopride and [^11^C]SCH23390 using Statistical Parametric Mapping (SPM 12) toolbox SAMIT (Small Animal Molecular Imaging Toolbox) (Vallez Garcia et al., 2015). Then, each dynamic image was coregistered to its radiotracer-specific template using PMOD 4.1 (PMOD Technologies LLC, Switzerland). Coregistered images were placed on a mouse brain atlas rescaled from Barriere et al. (2021), and time-activity curves (TAC) for each brain volume-of-interest (VOI) at each timepoint were extracted for kinetic analysis using the kinetic module from PMOD 4.1 (Janke and Ullmann, 2015).

### Kinetic Modeling

VOIs were placed bilaterally in CPu and NAc, reflecting dorsal and ventral striatum, respectively, and in cerebellum. The uptake over time from these VOIs was compared to uptake in cerebellum and kinetic analysis performed using the Simplified Reference Tissue Model (SRTM), SRTM2 and Reference Logan (Logan) to calculate binding potential (BP_*ND*_). For SRTM, the data is fitted in a non-linear fitting to obtain the values for tracer kinetics (Lammertsma and Hume, 1996). For SRTM2, a similar procedure is performed, but fixing k2′ values from the CPu analysis and applying the fixed value to the NAc (Wu and Carson, 2002). Lastly, a Reference Logan from the last 30 min of scan (t*30) was performed to obtain a linear estimation of the kinetic values (Logan et al., 1996). Akaike Information Criterion (AIC) showed that SRTM2 had slightly lower error estimates when compared to the other two kinetic models.

### Magnetic Resonance Imaging Acquisition

For structural MRI acquisition, animals were anesthetized using isoflurane (4% induction, 2% maintenance) and placed onto a cryocoil-specific MRI mouse bed using both tooth- and ear-bars to prevent head movement during MR scans. A subcutaneous bolus injection of medetomidine (Domitor^®^, Orion Pharma AB, Sweden. 0.05 mg/kg) was administered, after which the isoflurane concentration was steadily reduced from 2 to 0.5% over a course of 2 min. A constant infusion of medetomidine was provided at 0.1 mg/kg/h (*s.c.*), started 15 min after the initial bolus injection to maintain animals anesthetized. Mice were then positioned inside the MR scanner (Bruker BioSpec 94/20, running Paravision 6.0 software) with the brain in the center of the field-of-view. Animals were scanned using a T1 FLASH sequence (TR/TE: 50/8 ms; flip angle: 20) with 125 μm isotropic spatial resolution using a 1H Quadrature transmit/receive MRI cryogenic RF coil (MRI CryoProbe, Bruker, Germany) for signal reception. The total structural MRI scan time was 10 min. Two additional sequences were performed (Diffusion Tensor Imaging (DTI) and resting-state functional MRI (rs-fMRI) but will not be discussed in this study. Total scan duration was 90 min, but due to a large number of animals that died at 18 months, the duration of the scan was reduced to 60 min at 24-months to improve survival (Supplementary Figure 2). Throughout all MRI experiments, respiration and temperature were constantly monitored. After scanning, animals were administered 0.3 mL of 1:20 atipamezole (Atipam, Dechra Veterinary Products, Sweden) diluted in 0.9% saline to induce awakening and rehydration. Animals were placed into a recovery cage until fully awake, and then returned to its home cage.

### Preprocessing and Structural Magnetic Resonance Imaging Tissue Segmentation

Preprocessing of T1 images was performed using MATLAB-based SPM. Bias-corrected images had signal from outside of brain removed by applying an automated mask to individual scans. A study specific *in vivo* T1 template was generated in two coregistration steps. First, images of each animal were longitudinally coregistered to generate one averaged image from all time points of each subject. A second step created an age-balanced average of images generated from the first step to generate one study-specific template (*n* = 10).

For tissue segmentation, initial tissue class priors from Hikishima et al. (2017) were used. These were coregistered and resampled to our study-specific *in vivo* template space (0.125 mm isotropic) using the SPM8 toolbox, SPMmouse (Sawiak et al., 2013), followed by tissue segmentation toolbox in SPMmouse and DARTEL algorithm to generate preliminary tissue priors for our study (Ashburner, 2007). A second tissue segmentation and DARTEL step was performed using these preliminary tissue priors to obtain study- and sequence-specific tissue probability maps (TPMs). The resulting TPMs were used to segment images from each individual at 12, 18, and 24 months of age into gray matter, white matter, and cerebrospinal fluid (GM, WM, CSF, respectively) and used for voxel-based morphometric analysis. A second measurement of structural changes was performed using tensor-based morphometry (TBM) as described elsewhere (Asan et al., 2021), to determine local morphometric changes of brain structure with time.

### Regional Volumetric Changes

Masks for lateral ventricles and basal ganglia were manually created based from the age-balanced T1-weighted aging template (*n* = 11) using itk-SNAP (Yushkevich et al., 2006). These masks were the registered to individual timepoint scans for each individual to measure specific ventricle and basal ganglia volumes. A combination of block matching approach and free-form deformation methods is used for the registration, which is implemented in the NiftyReg (RRID:SCR_006593) open-source software. The resulting volumes were tested for significant correlation with time (age), with TIV was included as a fixed effect, with linear mixed effects statistical model using “lmer” package in R statistical software.

### Statistical Analysis

Data from animal body weight were evaluated for a general time-effect with Generalized Estimating Equation (GEE), with pairwise comparison between animals that underwent MRI only and animals with both PET and MRI during the 12 months. Both body weight over time and weight gain when compared to baseline body weight were assessed for both groups (Supplementary Figure 1).

Within-group BP_*ND*_ data obtained from VOI-based kinetic analysis in PMOD were assessed by Linear Mixed Effect (LME) model using R package lme4 (R Core Team, 1.1–23 Bates et al., 2015). The general effect of time was obtained for both dorsal (CPu) and ventral (NAc) striatum, and false discovery rate (FDR) correction for multiple comparisons was performed. A *p*-value lower than 0.05 was deemed statistically significant. For voxel-based morphometry of structural MRI, smoothed and modulated segmented images from each animal at each timepoint were tested for general time-effect with within-group using Linear Mixed Effects (LME) model. Modulated GM/WM density over time was modeled and tested for significance using lme4 (R Core Team, 1.1–23; Bates et al., 2015) with time and total intracranial volume as fixed effects and individuals as random effect. As this study focuses on the effect of aging on striatum, we used a custom-built striatal mask, based on Turone Mouse Brain Template Atlas (TMBTA) (Barriere et al., 2021), and manually corrected for the age-balanced T1-weighted aging template (*n* = 11) using itk-SNAP, to restrict analysis within this region. All data were corrected for false discovery rate (FDR) and a *p*-value lower than 0.05 was considered statistically significant.

For correlation between PET and MRI results, we performed a repeated-measure correlation for animals that had both PET and MRI. Two measurements were performed, using the BP_*ND*_ from the CPu: one with the average mGMD of the CPu, and another measurement with only the significant increases in mGMD over time. Due to the longitudinal nature of the data, a repeated-measure correlation was performed using the R package rmcorr, with *p*-value of 0.05 as considered statistically significant (Bakdash and Marusich, 2017).

## Results

### BP_*ND*_ Changes in Dopaminergic D_2_ Receptors, but Not D_1_, Over Time in Striatum

VOI analysis was performed by modeling the TAC of CPu and NAc, using three distinct reference-based kinetic models: SRTM, SRTM2, and Logan for both [^11^C]SCH23390 and [^11^C]Raclopride, with the TAC of cerebellum as reference tissue. AIC values indicated that, of the three kinetic models tested, SRTM2 fit best for all individuals at all timepoints, both for [^11^C]SCH23390 and for [^11^C]Raclopride. Hence, the data presented in this section will be those of the SRTM2 model. BP_*ND*_ data from SRTM and Logan are presented as Supplementary Figure 3.

**FIGURE 1:**
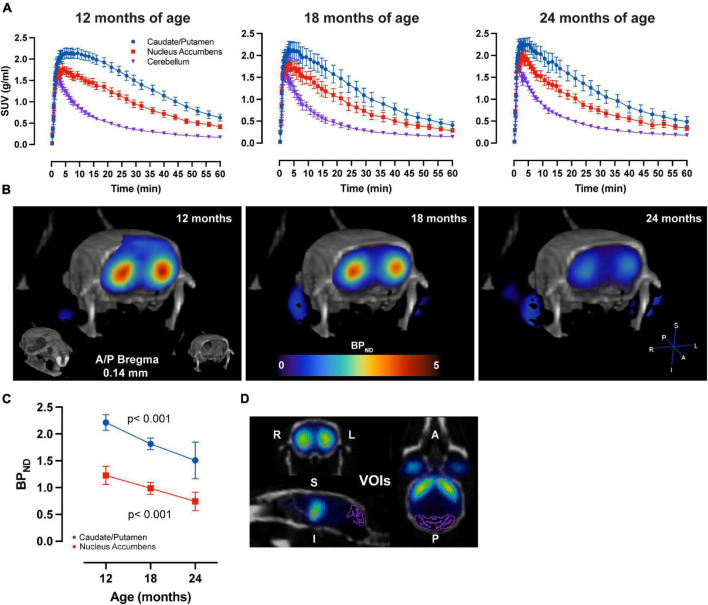
Aging-related D_2_ BP_*ND*_ changes. **(A)** Average ± SEM time-activity curves at 12, 18, and 24 months of age forstriatal regions and cerebellum. **(B)** Representative image of parametric BP_*ND*_ [^11^C]Raclopride image coregistered to CT of one animal at 12, 18, and 24 months of age. Colors represent BP_*ND*_ values from the PET image in striatum. **(C)** [^11^C]Raclopride BP_*ND*_ over time in CPu (blue) and NAc (red). LME data show a significant reduction over time (*p* < 0.001) for both CPu and NAc. **(D)** Representative image from PMOD demonstrating VOIs placement for the CPu (blue), NAc (orange) and cerebellum (purple) on an averaged PET image coregistered to CT.

**FIGURE 2:**
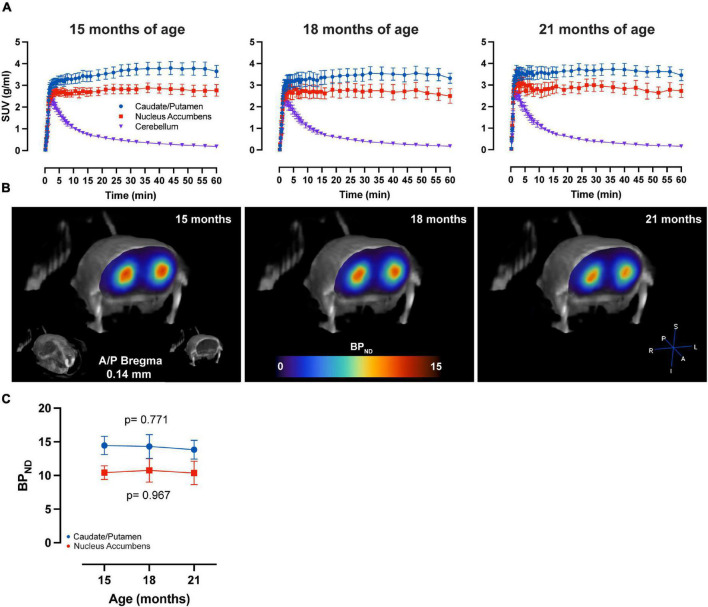
No age-related changes in D_1_ BP_*ND*_ over time. **(A)** Average ± SEM time-activity curves at 15, 18, and 21 months of age for striatal regions and cerebellum. **(B)** Representative image of parametric BP_*ND*_ [^11^C]SCH23390 image coregistered to CT of one animal at 15, 18, and 21 months of age. Colors represent BP_*ND*_ values from the PET image in the striatum. **(C)** [^11^C]SCH23390 BP_*ND*_ over time in CPu (blue) and NAc (red). LME data show no significant effect of time on CPu (*p* = 0.771) and NAc (*p* = 0.967).

LME analysis of SRTM2 models showed a highly significant time-effect for [^11^C]Raclopride in CPu (*t*-value: −4.628; *p* < 0.001) and NAc (*t*-value: −4.015; *p* < 0.001) (Figure 1). A linear trend for this decrease was observed in both CPu and NAc for [^11^C] Raclopride over 6 months (12.2 and 13.5% for CPu and NAc, respectively). VOI analysis of [^11^C]SCH23390 showed no time effect for either CPu (*t*-value: −0.299; *p* = 0.770; Figure 2) or NAc (*t*-value: 0.042; *p* = 0.967). The region with the most pronounced decrease in [^11^C]SCH23390 BP_*ND*_ was CPu, with a 2.83% decrease over 6 months, and NAc showed a slight increase (0.24%) over this time.

### Striatal Gray Matter Changes With Aging

**FIGURE 3:**
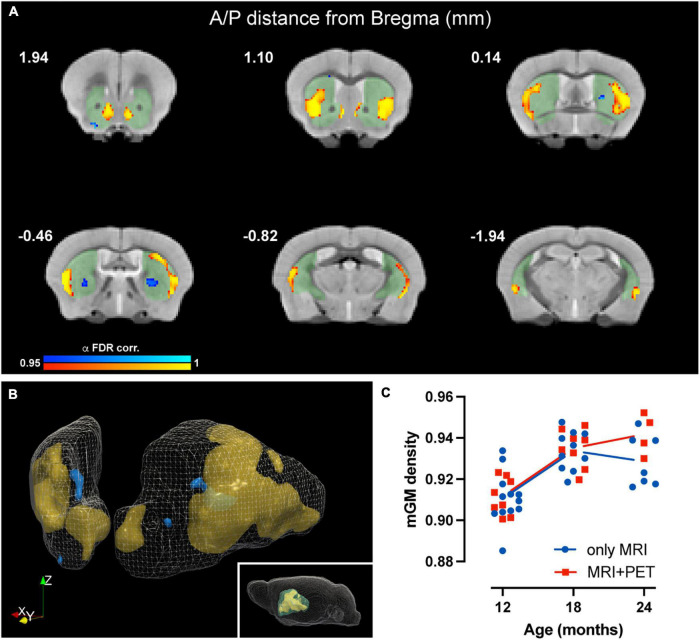
Age-related changes in modulated GM density in mouse striatum. **(A)** Coronal slices of structural MRI template depicting time-dependent decrease (blue) and increase (yellow) in mGMD (*p*-value after FDR-correction < 0.05). Striatal mask used for the analysis is shaded (green) to facilitate visualization. **(B)** Three-dimensional rendering of striatum, depicting decreases in mGMD in blue and increases in mGMD in orange. **(C)** Graphical representation of striatal clusters with increased mGMD over time. Each dot represents the mean mGMD of all clusters for each animal. Line represents overall mean values at each timepoint for both MRI only (blue) and MRI + PET (red) groups.

Voxel-based morphometry (VBM) analysis, sensitive to voxelwise changes in modulated gray matter density (mGMD), revealed age-dependent structural changes of gray matter in striatum. FDR-corrected within-group analysis using a linear mixed effects model revealed a significant effect of time, with a decrease in mGMD in nine small and discrete clusters located in posteromedial CPu and globus pallidus, as well as in lateral NAc, comprising a total of 216 voxels. Interestingly, a significant increase in mGMD was observed in four large clusters located in lateral CPu and in medial NAc, with a total of 3,221 voxels with significant differences over time (Figures 3A,B). These values correspond to 4.6 and 69.6% of the total number of voxels in striatum—or a volume of 0.42 and 6.3 mm^3^, respectively, either decreasing or increasing in GM density, respectively. These results, without FDR correction for multiple comparisons, are provided as Supplementary Figure 4. From significant clusters in striatum, the extracted mGMD values suggest that the observed increase over time is more pronounced between 12 and 18 months, while remaining relatively stable from 18 to 24 months (Figure 3C). In order to assess whether the amount of anesthesia could affect GM density over the course of the study, a linear mixed effect model comparing mGMD in animals that underwent MRI alone and animals with both MRI and PET scans was performed. As expected, a significant effect of aging was observed (*p* < 0.001), but with no significant differences found between groups (*p* = 0.233) nor an interaction between group and age (*p* = 0.093). FDR-corrected VBM of white matter (WM) yielded no significant difference over time (data not shown). Tensor based morphometry (TBM) maps, analyzed by LME, showed local shape changes (contraction) in cortical and hippocampal areas over time, in addition to subcortical changes (expansion) in septum, lateral ventricles and striatum (Supplementary Figure 5). Based upon these observations, a mask-based analysis of lateral ventricle and striatal volumes was performed. A general effect of time (age) was observed for basal ganglia volume (*t* = 4.804, *p* = 4.73e-05), which showed an increase over time, while total intercranial volume (TIV) showed a decrease (*t* = −3.567, *p* = 0.0009). When the effect of TIV is included as a covariate in the LME model, both lateral ventricle and basal ganglia volumes showed significant increases with age, *p* = 0.0339 and *p* = 3.62e-06, respectively.

### Dopamine D_2_ BP_*ND*_ in CPu Negatively Correlate With Significant GM Clusters With Aging

**FIGURE 4:**
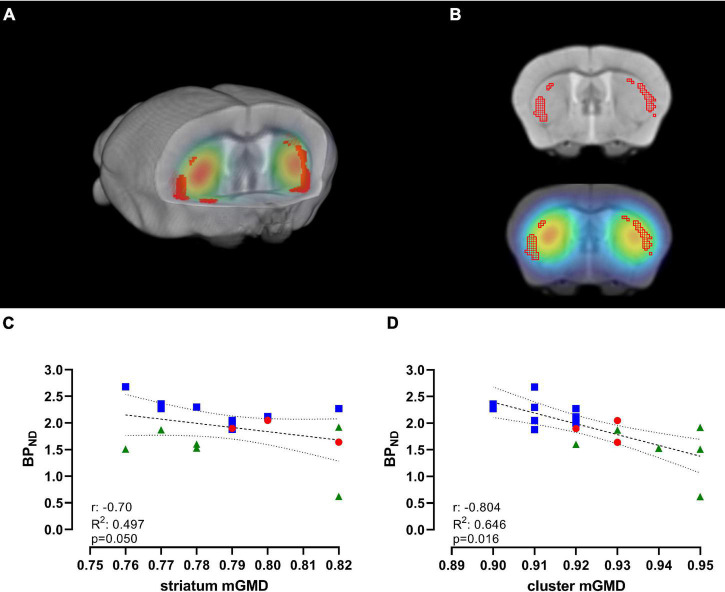
[^11^C]Raclopride BP_*ND*_ is negatively correlated with significant GM clusters, but not with CPu VOI. **(A)** 3D-rendering of skull-stripped structural MRI and coregistered [^11^C]Raclopride highlighting the CPu. Shaded regions in red represent clusters in the CPu that showed significant increase in mGMD over time. **(B)** Coronal slice of structural MRI (top) and coregistered PET/MRI (bottom) depicting the clusters with increase in mGMD (red mesh). Repeated-measure correlation between [^11^C]Raclopride BP_*ND*_ in the CPu and mGMD of the entire CPu **(C)** and restricted to the clusters that showed significant increase in mGMD **(D)**. Lines represent linear trend and 95% confidence interval. Blue squares, red circles and green triangles represent 12, 18, and 24 months, respectively.

As previously observed, we found a significant decrease in [^11^C]Raclopride with time, and significant changes in mGMD, in both directions, with a significant increase in GM density in CPu. To further understand the association of these changes over time, we performed a correlation between [^11^C]Raclopride and mGM density, within the entire striatal mask or limited to significant GM clusters for animals that underwent both [^11^C]Raclopride and structural MRI (Figures 4A,B; Bakdash and Marusich, 2017). There was a trend-level correlation between [^11^C]Raclopride BP_*ND*_ and striatal mGMD (*r* = −0.705, *R*^2^ = 0.497, *p* = 0.050; Figure 4C). However, when comparing [^11^C]Raclopride BP_*ND*_ against clusters that showed significant increases in VBM, a significant correlation was observed between the BP_*ND*_ and mGMD (*r* = −0.804, *R*^2^ = 0.646, *p* = 0.016; Figure 4D).

## Discussion

Longitudinal imaging of aging animals across the entire lifespan is a promising tool to complement human aging studies. This study was aimed to explore age-related changes in dopamine D_1_ and D_2_ system in mice in a longitudinal setting. Additionally, we also aimed to observe age-related changes in striatal morphometry using longitudinal structural MRI. Our findings suggest that dopaminergic D_2_ receptors dramatically change during normal aging, as indicated by a steep reduction in [^11^C]Raclopride BP_*ND*_ in striatum—especially in CPu over a 12-month period. sMRI through VBM analysis revealed significant increases and decreases over the same period, irrespective of total volume, as assessed by TBM. The increase in mGMD found in dorsal striatum significantly correlated with decreased [^11^C]Raclopride BP_*ND*_. Interestingly, no changes were observed in D_1_ BP_*ND*_ over a 6-month period measured by [^11^C]SCH23390 PET imaging. This is the first time a longitudinal, long-term approach was used in aged mice in a multimodal fashion to understand the consequence of senescence on the dopaminergic system in mouse striatum.

### Changes in [^11^C]Raclopride, but Not [^11^C]SCH23390, BP_*ND*_ Over Time

PET imaging of dopamine receptors has been widely used to understand changes in receptor behavior over time. In humans, [^11^C]Raclopride BP_*ND*_ decreases during senescence, ranging from 5 to 10% per decade (Volkow et al., 1998; Ishibashi et al., 2009; Karrer et al., 2017). Most studies attempting to address dopaminergic decline during aging in humans report such decrease using cross-sectional studies, although a great effort has been made to evaluate age-related changes in the human dopaminergic system in recent years (Nevalainen et al., 2015). Consistent with human work and cross-sectional *in vitro* estimates in rats (O’Boyle and Waddington, 1984; Suzuki et al., 2001; Hoekzema et al., 2010), we observed a significant reduction in [^11^C]Raclopride BP_*ND*_ over the course of 12 months in mice (i.e., from 12 to 24 months of age). A linear regression revealed that the BP_*ND*_ decrease was around 11–12% every 6 months, dependent upon the region assessed. This 6-month interval is roughly 15–20 years in human age. Thus, this study would roughly encompass a longitudinal assessment of a human subject from 40 until 80 years of age (Wang et al., 2020). Collectively then, cross-sectional and longitudinal, *in vivo* and *in vitro*, assessment of D_2_ follow a similar trend: a decrease of receptor in striatum with age. Our findings indicate that longitudinal PET imaging of D_2_ in animal models can be related, to a certain extent, to what is observed in human senescence, and animal models can be used to understand the molecular pathways of dopamine D_2_ receptor in health, and likely how alterations on dopaminergic neuromodulation can possibly affect aging. This finding opens up for new avenues for future research in which longitudinal imaging of D_2_ receptors in mice may be used to further study the D_2_ systems as a mediator of age-related changes in brain function and behavior at a fraction of time compared to humans.

Interestingly, our data indicate that there was no significant effect of time on [^11^C]SCH23390 BP_*ND*_ in striatum over the course of 6 months (i.e., from 15 to 21 months of age). These findings are contrary to studies in humans that report a significant age-related difference in D_1_ BP_*ND*_ over time in both caudate and putamen (Backman et al., 2011; Rieckmann et al., 2011; Karrer et al., 2017). Animal studies report variable results regarding the effect of senescence on D_1_ receptor BP_*ND*_ in striatum (O’Boyle and Waddington, 1984; Suzuki et al., 2001). These findings, together with our results, suggest that senescence may influence D_1_ receptors differently between rodents and humans, or that the imaging technique itself is not readily translatable between species.

The main hurdle for a direct translation between human and animal studies involves the use of general anesthetics. Isoflurane, the most widely used substance for the induction and maintenance of general anesthesia in rodents, is reported to modulate striatal γ-aminobutyric acid (GABA) transmission and dopamine release in striatum (Adachi et al., 2005; Liu et al., 2020), which may influence dopamine release and its subsequent binding to dopamine receptors. In fact, it is known that D_1_ receptors respond in a more phasic manner than D_2_, thus requiring a larger amount of dopamine to elicit D_1_- dependent response (Suzuki et al., 2001; Dreyer et al., 2010). Our results with [^11^C]SCH23390 shows that the TAC of the radiotracer has a slower washout in receptor-rich regions compared to [^11^C]Raclopride, suggesting that displacement of this D_1_ antagonist (SCH23390) by dopamine occurs slowly over time (Suzuki et al., 2001; Alstrup et al., 2013). It is possible that anesthesia limits DA release from happening, resulting in fewer dopamine bursts while anesthetized. This greatly reduces the possibility of translational assessment of [^11^C]SCH23390 as a surrogate of D_1_ receptor availability, and animal studies using such tracer should take great care in the interpretation of findings derived from this radiotracer. A possible method for future assessment of whether anesthesia influences [11C]SCH23390 TAC in mice is to administer radiotracer to awake animals prior to a static scan (e.g., a 30-min scan, 60 min after tracer administration). This will allow for a comparison of radioligand uptake in striatum of anesthetized animals to those that were awake between the administration of tracer and PET.

### Changes in Modulated Gray Matter Density in Striatum of Aging Mice

Analysis of the striatum over time showed significant morphometric changes in TBM, changes in mGMD measured by VBM, with areas of CPu and NAc demonstrating increases and decreases in mGMD and an increase in striatal and lateral ventricle volumes. Interestingly, mGMD primarily increased in striatum while only a few significant clusters showed significant decreases in mGMD over time. Many studies report cortical thickness decrease over time in humans, and such decreases are often associated with age-related cognitive decline (Giorgio et al., 2010; Terribilli et al., 2011; Ramanoel et al., 2018; Irimia, 2021). Studies with focus placed on subcortical structures—such as basal ganglia—are limited and report varied results (Raz et al., 2003; Pfefferbaum et al., 2013; Tullo et al., 2019). In rodents, VBM has demonstrated similar trends to that reported in human studies. Many studies regarding aging in rodents (mice and rats) primarily focus on cortical structures and somehow neglect subcortical regions of the brain, such as basal ganglia (Hammelrath et al., 2016; Taylor et al., 2020; Asan et al., 2021). Our structural data points toward changes in striatum over time, with bilateral increases in mGMD observed in lateral portions of dorsal striatum, medial portions of nucleus accumbens, and bilateral reduction in mGMD in globus pallidus. One seminal study from Han et al. (1989) using immunohistochemistry showed that neuronal cells in rat striatum decrease 19% in density, yet the total density of striatum is increased 13.5% when comparing 6-month-old against 24-month-old animals. The increase found by the authors is mostly due to a 58% increase in non-neuronal cells (Han et al., 1989). In fact, senescence effects glia as well as neurons, with astrocytes and microglia showing hypertrophic and hyperactivated states with aging both in animals (Duncombe et al., 2017; Ashraf et al., 2019) and humans (Verkerke et al., 2021).

Interestingly we found in our study a similar pattern, with a correlation between decreases in D_2_ BP_*ND*_ and mGMD increase, suggesting an association between decrease in [^11^C]Raclopride over time—indicating a loss of neuronal function over time—and changes to mGMD—possibly indicating an increased number of non-neuronal cells. Thus, it is possible that the increase in mGMD found by this study is due to increases in glia, as is already observed in natural aging. On the other hand, some voxels indicate decreases in mGMD, suggesting that changes in the senescent striatum are not uniform, as expected from a structure involved in many different functions. In order to confirm such hypothesis, future studies on the matter should ally both longitudinal and cross-sectional, immunohistochemical approaches to confirm our findings.

This study is not without its limitations. First, even though the longitudinal assessment adds a significant improvement to evaluate differences in dopamine receptor BP_*ND*_ over a 12-month course of aging, the small number of animals for the PET assessment poses the main challenge, especially when considering a 12-month follow-up study design. In addition, a considerable number of animals did not survive to the end of the experiment, contributing to the larger variability observed at later timepoints. Another limitation is the number of times and duration of anesthesia procedures per animal, especially when considering the differences between mice that had only MRI and mice that had both PET and MRI scans. Animals with PET and MRI had three times more anesthesia when compared with their MRI only counterparts. We suggest future longitudinal studies using very old animals to maintain anesthesia duration to a maximum of 60 min, and repeated scan procedures to be performed on separate days in order to give time for animals to recover. Another limitation for this study is the lack of cross-sectional, immunohistochemical data for dopamine-related signaling, such as dopamine D_1_ and D_2_ receptors, as well as for the putative increase in non-neuronal cells as the cause of increased mGMD over time. We suggest that future studies, with the aim to further understand the dopamine system over the course of aging, include subsets of animals to be evaluated at specific timepoints to provide cross-sectional validation of what is observed in PET and MRI over time.

In conclusion, we found that D_2_ receptor BP_*ND*_ in striatum decreased over the course of 12 months as measured with [^11^C]Raclopride. These data are generally in line with cross-sectional reports in humans and encourage longitudinal work in mice as a complementary tool to study age-related changes in DA receptors and its causes and consequences. D_1_ BP_*ND*_—using [^11^C]SCH23390—remained constant, which we believe reflects an effect of the anesthesia and thus discourages [^11^C]SCH23390 as a translational biomarker of brain aging in mice. We also found a significant effect of time for structural gray matter changes in striatum, displaying both increases and decreases throughout the structure, with a significant correlation between increases in mGMD and decreases in D_2_ receptor availability during senescence. These data suggest that, at least for [^11^C]Raclopride, it is feasible to perform aging studies in mice to further understand the basic mechanisms of D_2_ receptors with age. However, additional studies for D_1_ are required to better understand which mechanisms are at play for the overall lack of significant decreases for this receptor over time. Further research is needed to understand the unexpected increase in mGMD in striatum and determine whether this is indeed due to an increase in non-neuronal cells.

## Data Availability Statement

The raw data supporting the conclusions of this article will be made available by the authors, without undue reservation.

## Ethics Statement

The animal study was reviewed and approved by the Animal Research Ethics Committee of Northern Norrland and by the Swedish Board of Agriculture (permit: A17-2019).

## Author Contributions

BG, AR, FS, and DM designed the research. BG, TM, ME, FS, and DM performed the research. BG, ÖÖ, JA, FS, and DM analyzed the data. BG and DM wrote the manuscript. BG, ÖÖ, TM, ME, JA, AR, FS, and DM contributed to the revision and approved the final submission. All authors contributed to the article and approved the submitted version.

## Conflict of Interest

The authors declare that the research was conducted in the absence of any commercial or financial relationships that could be construed as a potential conflict of interest.

## Publisher’s Note

All claims expressed in this article are solely those of the authors and do not necessarily represent those of their affiliated organizations, or those of the publisher, the editors and the reviewers. Any product that may be evaluated in this article, or claim that may be made by its manufacturer, is not guaranteed or endorsed by the publisher.
