# Supplementary material for: The Aged Striatum: Evidence of Molecular and Structural Changes Using a Longitudinal Multimodal Approach in Mice
Source: Front Aging Neurosci. 2022 Jan 24;14:795132. doi: 10.3389/fnagi.2022.795132 (PMC8818755; doi:10.3389/fnagi.2022.795132)
Supplement: Supplementary file 1 [file Table_1.DOCX]

Supplementary Material

# Supplementary Data

# Supplementary Figures and Tables

## Supplementary Figures

**
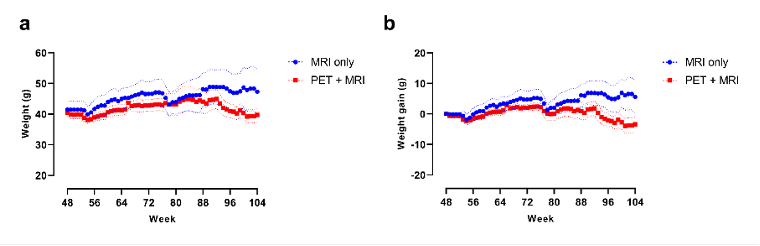
**

**Supplementary Figure S1.** Weight changes over time. Animals were weighed weekly from six months-old until the end of the experiments, at 24 months-old. (**A**) weight (in grams) over time. GEE analysis showed a significant main effect of time (p<0.001), as well as a significant effect of group (p=0.043) and time vs. group interaction (p<0.001). (**B**) changes in weight (in grams) when compared to baseline

**
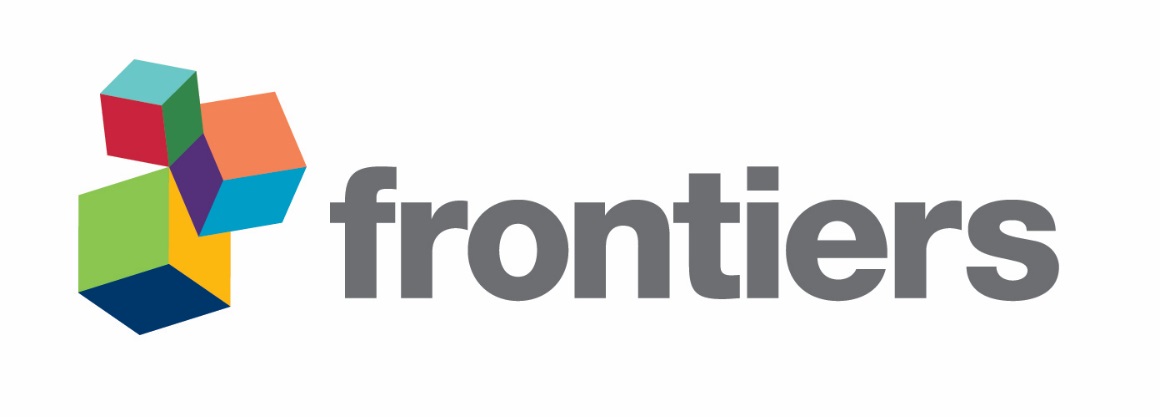
**

**
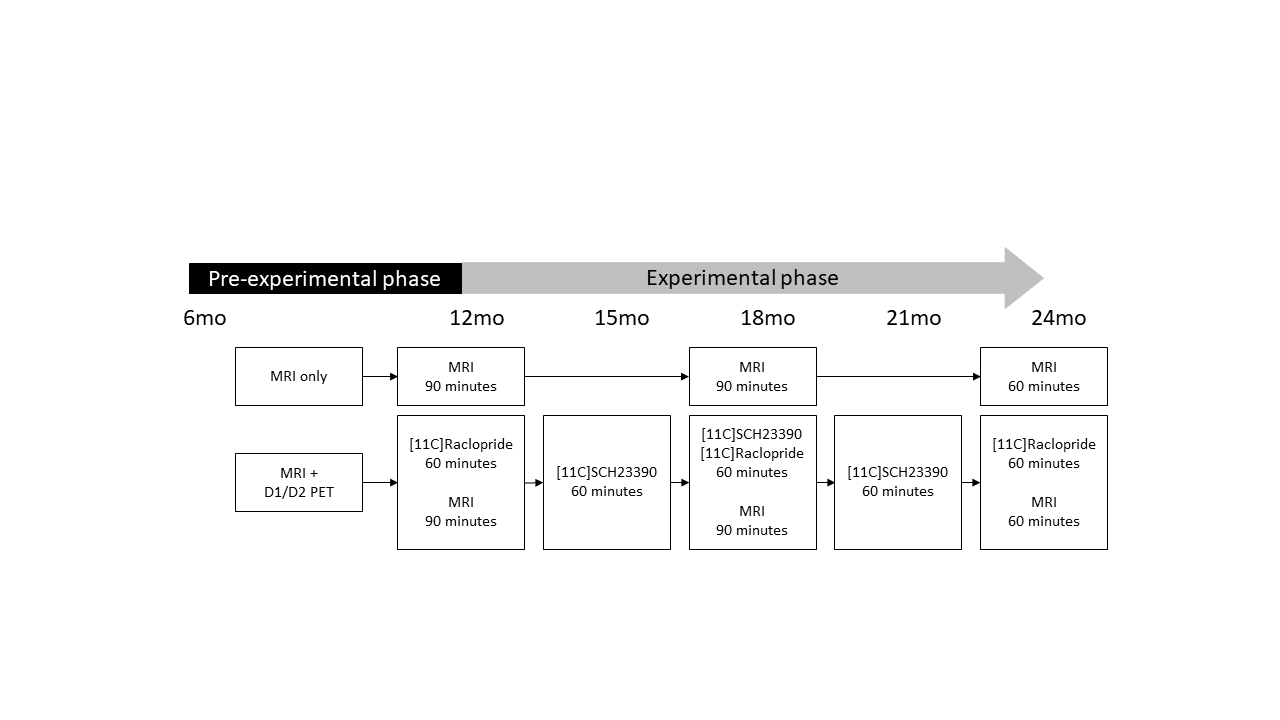
**

**Supplementary Figure S2.** Experimental design. 6-month-old mice were allowed to age until reaching 12 months of age before initiating longitudinal MRI and PET imaging (pre-experimental phase). At 12 months of age (Experimental phase), animals were longitudinally observed during 12 months using MRI scans scheduled every sixth month (MRI only group). Additionally, a subset of animals was also submitted to PET scans for D_1_- and D_2_-receptor imaging using [^11^C]SCH23390 and [^11^C]Raclopride, respectively (MRI + D_1_/D_2_ PET group). A complete table with sample size at all timepoints can be found in Supplementary Material, Table S1

**
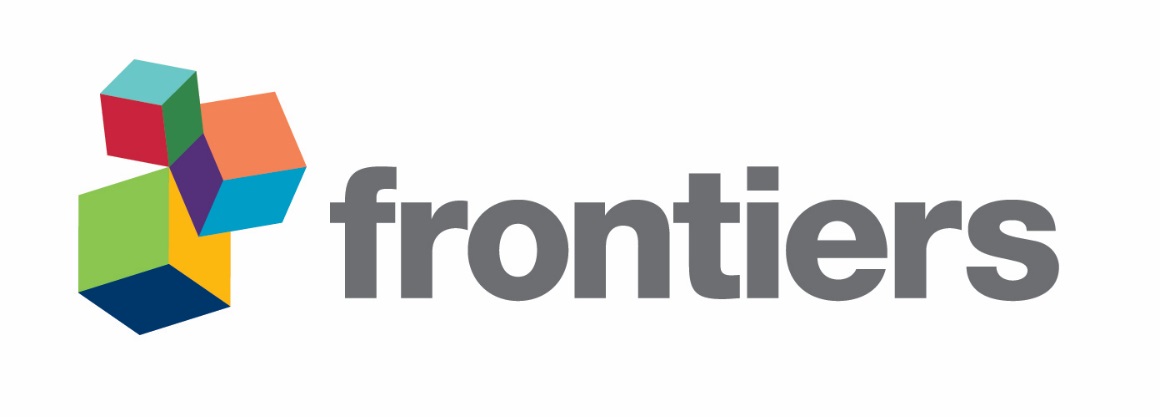
**


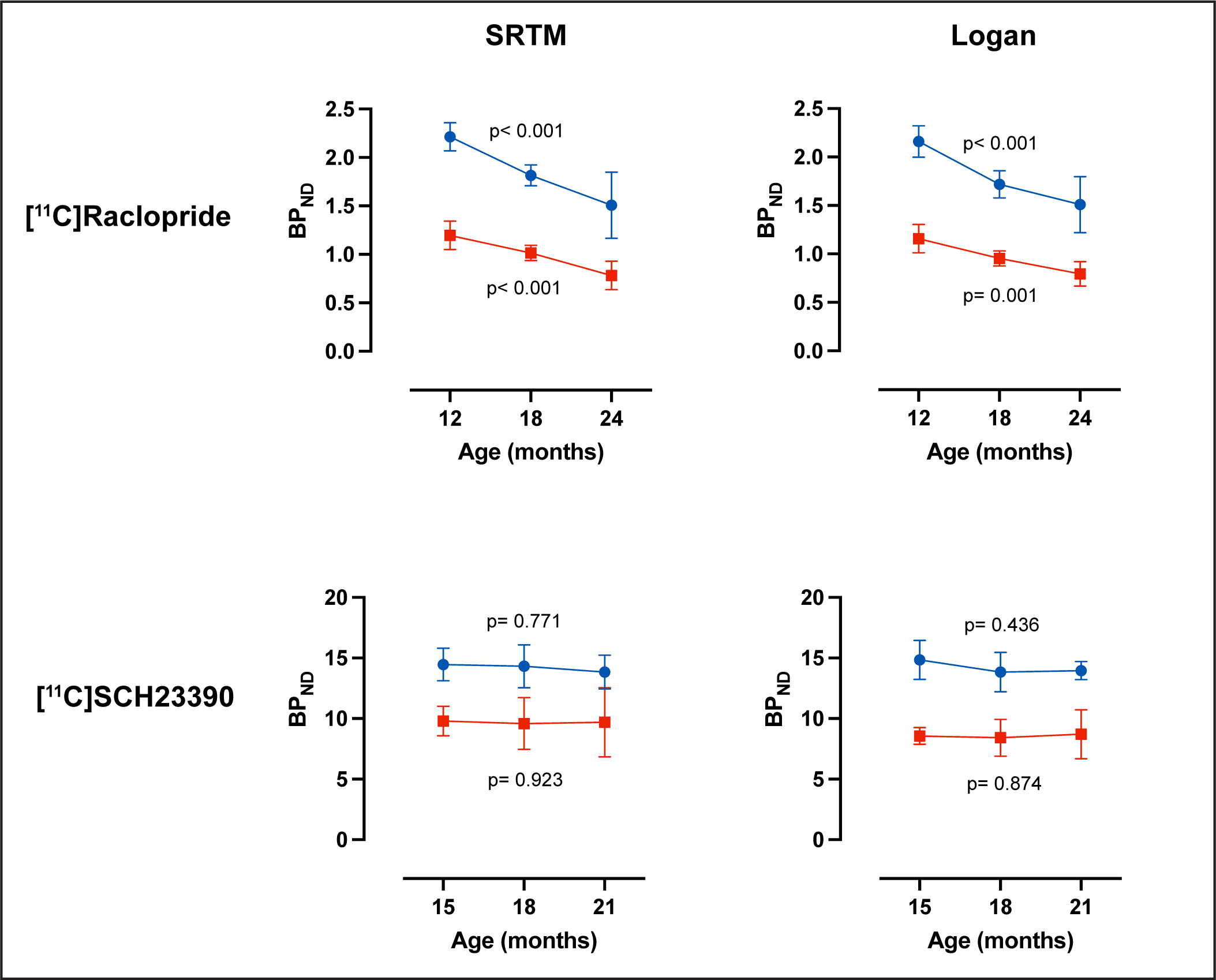


**Supplementary Figure S3.** Kinetic models from [^11^C]Raclopride (top row) and [^11^C]SCH23390 (bottom row) over time using SRTM (left column) and Logan (right column) kinetic models. CPu and NAc are represented in blue circles and red squares, respectively. LME show a significant reduction over time in both CPu and NAc for [^11^C]Raclopride and no significant effect of time for [^11^C]SCH23390, for which the p-values for the general effect of time (age) for each striatal region are indicated

**
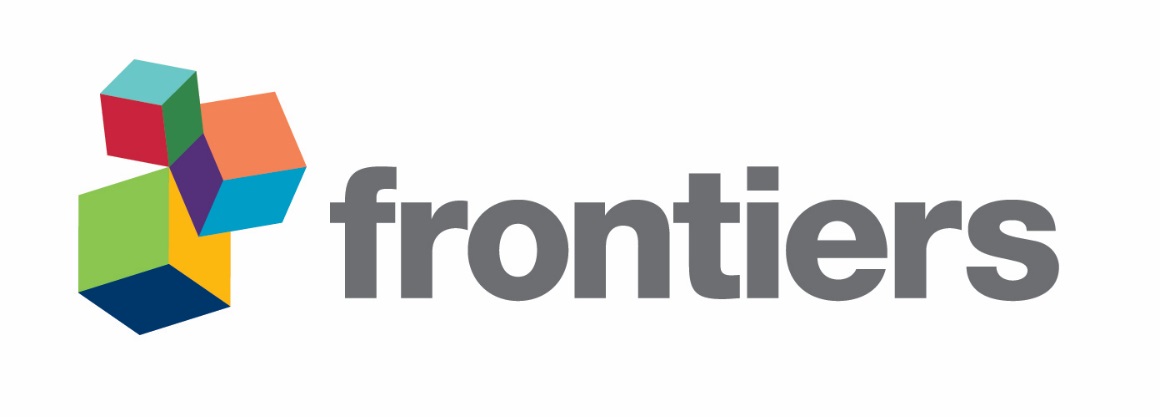
**


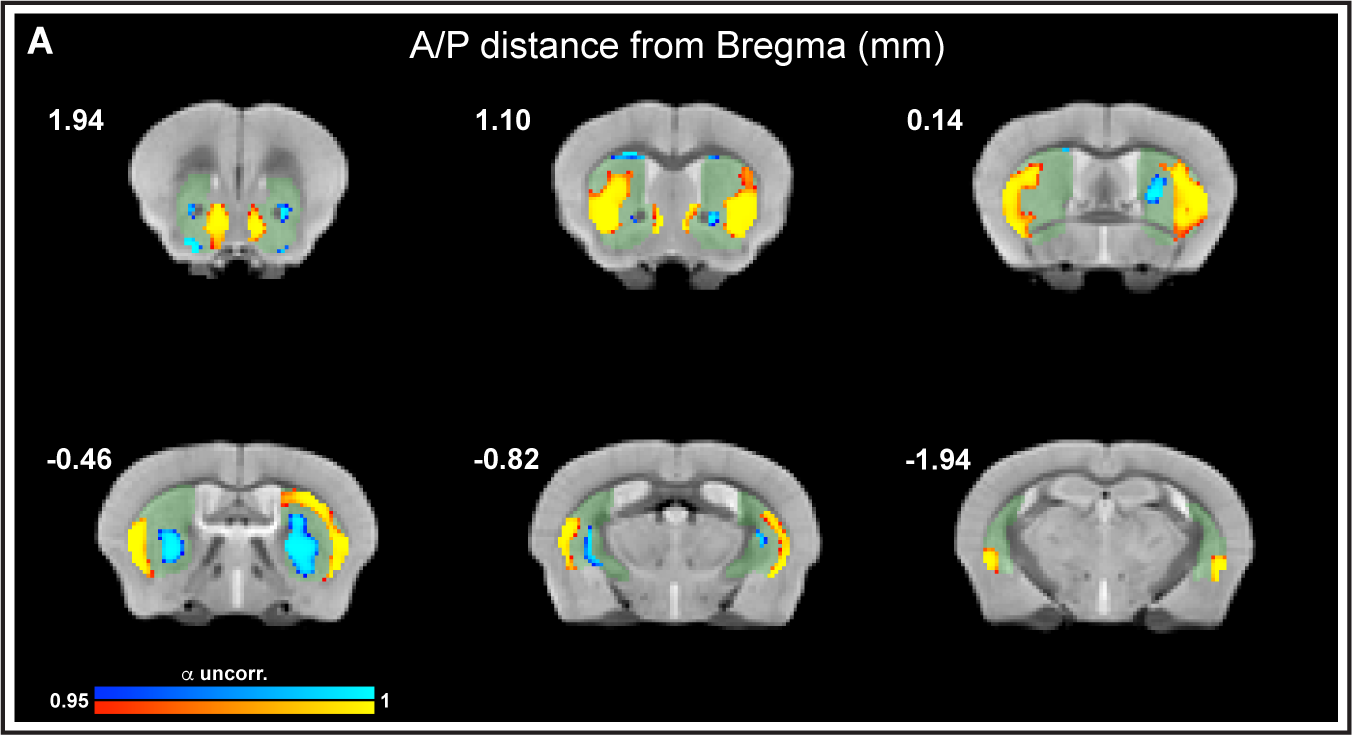


**Supplementary Figure S4.** Age-related changes in modulated GM density in mouse striatum without FDR correction for multiple comparisons. (**A**) coronal slices of structural MRI template depicting time-dependent decrease (blue) and increase (yellow) in mGMD (p < 0.05, without FDR correction for multiple comparisons). Striatal mask used for the analysis is shaded (green) to facilitate its visualization


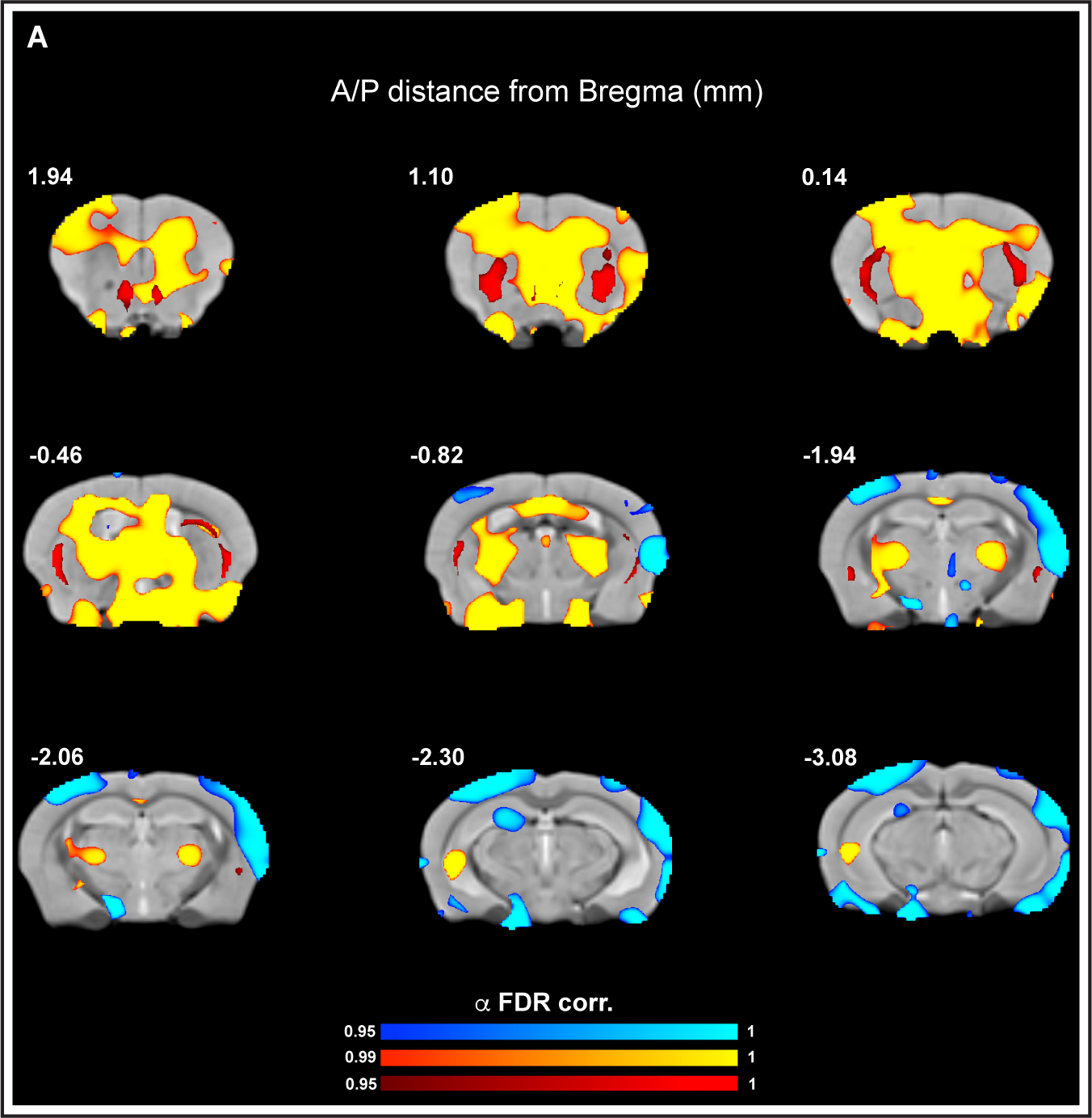


**Supplementary Figure S5:** Voxelwise maps of estimated age-related local morphometric changes of brain structure using TBM. (**A**) coronal slices of the study-specific structural MRI template together with the statistical maps depicting brain areas exhibiting significant volume growth or volume loss. Time-dependent decrease (blue, p < 0.05 after FDR correction for multiple comparisons) and increase (yellow; p < 0.01 after FDR correction) in different brain areas. The time-dependent increase (red, p < 0.05 after FDR correction) in mGMD are also superimposed to indicate the difference in location between morphometric changes observed by TBM and the significant increases in mGMD observed by VBM

## Supplementary Tables

Table S1: Study sample size

| Animal  PET-MRI | 12 months | | 15 months | 18 months | | | 21 months | 24 months | |
| --- | --- | --- | --- | --- | --- | --- | --- | --- | --- |
|  | **MRI** | **D_2_-PET** | **D_1_-PET** | **MRI** | **D_1_-PET** | **D_2_-PET** | **D_1_-PET** | **MRI** | **D_2_-PET** |
| 1 | **X** | **X** | **X** | **X** | **X** | **X** | **X** | **X** | **X** |
| 2 | **X** | **X** | **X** | **X** | **X** | **X** | **X** | **X** | **X** |
| 3 | **X** | **X** | **X** | **X** | **X** | **X** | **X** | **X** | **X** |
| 4 | **X** | **X** | **X** | **X** | **X** | ***** | **X** | **X** | **X** |
| 5 | **X** | **X** | **X** | **X** | **X** | **-** | **-** | **-** | **-** |
| 6 | **X** | **X** | **X** | **X** | **-** | **-** | **-** | **-** | **-** |
| 7 | **X** | **X** | **X** | **-** | **-** | **-** | **-** | **-** | **-** |
| 8 | **X** | **X** | **X** | **-** | **-** | **-** | **-** | **-** | **-** |
| 9 | **X** | **X** | **-** | **-** | **-** | **-** | **-** | **-** | **-** |
|  |  |  |  |  |  |  |  |  |  |
| Animal  MRI only | 12 months | | 15 months | 18 months | | | 21 months | 24 months | |
|  | **MRI** | **D_2_-PET** | **D_1_-PET** | **MRI** | **D_1_-PET** | **D_2_-PET** | **D_1_-PET** | **MRI** | **D_2_-PET** |
| 10 | **X** |  |  | **X** |  |  |  | **X** | **X** |
| 11 | **X** |  |  | **X** |  |  |  | **X** | **X** |
| 12 | **X** |  |  | **X** |  |  |  | **X** |  |
| 13 | **X** |  |  | **X** |  |  |  | **X** |  |
| 14 | **X** |  |  | **X** |  |  |  | **X** |  |
| 15 | **X** |  |  | **X** |  |  |  | **X** |  |
| 16 | **X** |  |  | **X** |  |  |  | **X** |  |
| 17 | **X** |  |  | **X** |  |  |  | **-** |  |
| 18 | **X** |  |  | **X** |  |  |  | **-** |  |
| 19 | **X** |  |  | **X** |  |  |  | **-** |  |
| 20 | **X** |  |  | **X** |  |  |  | **-** |  |
| 21 | **X** |  |  | **X** |  |  |  | **-** |  |
| 22 | **X** |  |  | **-** |  |  |  | **-** |  |
|  |  |  |  |  |  |  |  |  |  |
| Animal  PET only | 12 months | | 15 months | 18 months | | | 21 months | 24 months | |
|  | **MRI** | **D_2_-PET** | **D_1_-PET** | **MRI** | **D_1_-PET** | **D_2_-PET** | **D_1_-PET** | **MRI** | **D_2_-PET** |
| 23 |  |  |  |  |  | **X** |  |  |  |
| 24 |  |  |  |  |  | **X** |  |  |  |
| 25 |  |  |  |  |  | **X** |  |  |  |
| **-** | **animal died** | |  |  |  |  |  |  |  |
| ***** | **failed scan** | |  |  |  |  |  |  |  |
| **X** | **successful scan** | |  |  |  |  |  |  |  |

Table S2: Descriptive binding potential (BP_ND_) for Caudate/Putamen and Nucleus Accumbens for each kinetic model. Data is presented as mean BP_ND_ (95%CI).

| **Raclopride** | **Caudate/Putamen** | | | **Nucleus accumbens** | | |
| --- | --- | --- | --- | --- | --- | --- |
|  | SRTM | SRTM2 | Logan ref. | SRTM | SRTM2 | Logan ref. |
| 12 m.o. | 2.213  (2.002 - 2.424) | 2.213  (2.002 - 2.424) | 2.16  (1.956 - 2.364) | 1.197  (1.057 - 1.336) | 1.228  (1.062 - 1.394) | 1.158  (1.024 - 1.292) |
| 18 m.o. | 1.815  (1.556 - 2.074) | 1.815  (1.556 - 2.074) | 1.718  (1.468 - 1.968) | 1.015  (0.844 - 1.186) | 0.987  (0.784 - 1.19) | 0.954  (0.789 - 1.118) |
| 24 m.o. | 1.508  (1.249 - 1.767) | 1.508  (1.249 - 1.767) | 1.509  (1.259 - 1.759) | 0.783  (0.612 - 0.954) | 0.743  0.54 - 0.946) | 0.795  (0.63 - 0.959) |
| **SCH23390** | **Caudate/Putamen** | | | **Nucleus accumbens** | | |
|  | SRTM | SRTM2 | Logan ref. | SRTM | SRTM2 | Logan ref. |
| 15 m.o. | 14.464  (12.91 - 16.01) | 14.464  (12.91 - 16.01) | 14.843  (13.24 - 16.44) | 9.803  (7.988 - 11.617) | 10.429  (9.009 - 11.848) | 8.563  (7.283 - 9.842) |
| 18 m.o. | 14.316  (12.35 - 16.28) | 14.316  (12.35 - 16.28) | 13.834  (11.81 - 15.85) | 9.588  (7.293 - 11.883) | 10.766  (8.97 - 12.562) | 8.414  (6.795 - 10.033) |
| 21 m.o. | 13.848  (11.65 - 16.045) | 13.848  (11.65 - 16.04) | 13.963  (11.70 - 16.22) | 9.693  (6.731 - 12.656) | 10.383  (8.375 - 12.39) | 8.705  (6.895 - 10.515) |
|  |  |  |  |  |  |  |
